# Retraction Note: Antibodies against endogenous retroviruses promote lung cancer immunotherapy

**DOI:** 10.1038/s41586-026-10104-7

**Published:** 2026-01-14

**Authors:** Kevin W. Ng, Jesse Boumelha, Katey S. S. Enfield, Jorge Almagro, Hongui Cha, Oriol Pich, Takahiro Karasaki, David A. Moore, Roberto Salgado, Monica Sivakumar, George Young, Miriam Molina-Arcas, Sophie de Carné Trécesson, Panayiotis Anastasiou, Annika Fendler, Lewis Au, Scott T. C. Shepherd, Carlos Martínez-Ruiz, Clare Puttick, James R. M. Black, Thomas B. K. Watkins, Hyemin Kim, Seohee Shim, Nikhil Faulkner, Jan Attig, Selvaraju Veeriah, Neil Magno, Sophia Ward, Alexander M. Frankell, Maise Al Bakir, Emilia L. Lim, Mark S. Hill, Gareth A. Wilson, Daniel E. Cook, Nicolai J. Birkbak, Axel Behrens, Nadia Yousaf, Sanjay Popat, Allan Hackshaw, TRACERx Consortium, Kevin W. Ng, Katey S. S. Enfield, Oriol Pich, Takahiro Karasaki, David A. Moore, Roberto Salgado, Monica Sivakumar, Carlos Martínez-Ruiz, Clare Puttick, James R. M. Black, Thomas B. K. Watkins, Selvaraju Veeriah, Sophia Ward, Alexander M. Frankell, Maise Al Bakir, Emilia L. Lim, Mark S. Hill, Gareth A. Wilson, Nicolai J. Birkbak, Allan Hackshaw, Andrew Rowan, Ariana Huebner, Brittany B. Campbell, Chris Bailey, Claudia Lee, Dhruva Biswas, Emma Colliver, Foteini Athanasopoulou, Haoran Zhai, Jayant K. Rane, Kristiana Grigoriadis, Michelle Dietzen, Michelle Leung, Mihaela Angelova, Olivia Lucas, Othman Al-Sawaf, Rachel Rosenthal, Jerome Nicod, Abigail Bunkum, Antonia Toncheva, Christopher Abbosh, Corentin Richard, Cristina Naceur-Lombardelli, Francisco Gimeno-Valiente, Jie Min Lam, Kerstin Thol, Krupa Thakkar, Mariana Werner Sunderland, Martin D. Forster, Nnennaya Kanu, Paulina Prymas, Robert Bentham, Sadegh Saghafinia, Sergio A. Quezada, Sharon Vanloo, Simone Zaccaria, Siow Ming Lee, Sonya Hessey, Wing Kin Liu, Dionysis Papadatos-Pastos, James Wilson, Sarah Benafif, Tanya Ahmad, Elaine Borg, Mary Falzon, Reena Khiroya, Teresa Marafioti, Abigail Sharp, Camilla Pilotti, Harjot Kaur Dhanda, Kitty Chan, Nicole Gower, Rachel Leslie, Sean Smith, Andrew G. Nicholson, Eric Lim, Javier Herrero, Carla Castignani, Elizabeth Larose Cadieux, Jonas Demeulemeester, Peter Van Loo, Karl S. Peggs, Catarina Veiga, Gary Royle, Charles-Antoine Collins-Fekete, Alexander James Procter, Arjun Nair, Asia Ahmed, Magali N. Taylor, Neal Navani, Ricky M. Thakrar, David Lawrence, Davide Patrini, Emma Nye, Richard Kevin Stone, David Chuter, Mairead MacKenzie, Francesco Fraioli, Paul Ashford, Sam M. Janes, Miljana Tanić, Stephan Beck, Alexandra Rice, Anand Devaraj, Chiara Proli, Daniel Kaniu, Harshil Bhayani, Hema Chavan, Hilgardt Raubenheimer, Lyn Ambrose, Mpho Malima, Nadia Fernandes, Paulo De Sousa, Pratibha Shah, Sarah Booth, Silviu I. Buderi, Simon Jordan, Sofina Begum, Ekaterini Boleti, Aengus Stewart, Alastair Magness, Clare E. Weeden, Dina Levi, Eva Grönroos, Jacki Goldman, Mickael Escudero, Philip Hobson, Roberto Vendramin, Stefan Boeing, Tamara Denner, Vittorio Barbè, Wei-Ting Lu, William Hill, Yutaka Naito, Zoe Ramsden, Anca Grapa, Hanyun Zhang, Khalid AbdulJabbar, Xiaoxi Pan, Kayleigh Gilbert, Angeliki Karamani, Benny Chain, David R. Pearce, Despoina Karagianni, Elena Hoxha, Felip Gálvez-Cancino, Georgia Stavrou, Gerasimos Mastrokalos, Helen L. Lowe, Ignacio Matos, James L. Reading, John A. Hartley, Kayalvizhi Selvaraju, Kezhong Chen, Leah Ensell, Mansi Shah, Marcos Vasquez, Maria Litovchenko, Olga Chervova, Piotr Pawlik, Robert E. Hynds, Saioa López, Samuel Gamble, Seng Kuong Anakin Ung, Supreet Kaur Bola, Thanos P. Mourikis, Victoria Spanswick, Yin Wu, Emilie Martinoni Hoogenboom, Fleur Monk, James W. Holding, Junaid Choudhary, Kunal Bhakhri, Marco Scarci, Martin Hayward, Nikolaos Panagiotopoulos, Pat Gorman, Robert C. M. Stephens, Steve Bandula, Yien Ning Sophia Wong, Tristan Clark, Heather Cheyne, Mohammed Khalil, Shirley Richardson, Tracey Cruickshank, Babu Naidu, Gurdeep Matharu, Jacqui A. Shaw, Joan Riley, Lindsay Primrose, John Le Quesne, Kevin G. Blyth, Alastair Kerr, Alexandra Clipson, Anshuman Chaturvedi, Caroline Dive, Dominic G. Rothwell, Elaine Kilgour, Jonathan Tugwood, Lynsey Priest, Pedro Oliveira, Philip Crosbie, Gillian Price, Judith Cave, Keith M. Kerr, Colin R. Lindsay, Fiona H. Blackhall, Matthew G. Krebs, Yvonne Summers, Alan Kirk, Mathew Thomas, Mo Asif, Nikos Kostoulas, Rocco Bilancia, Gary Middleton, Michael J. Shackcloth, Angela Leek, Jack Davies Hodgkinson, Nicola Totten, Craig Dick, Lily Robinson, Peter Russell, Madeleine Hewish, Sarah Danson, Jason F. Lester, Fabio Gomes, Kate Brown, Mathew Carter, Akshay J. Patel, Aya Osman, Christer Lacson, Gerald Langman, Helen Shackleford, Madava Djearaman, Salma Kadiri, Aiman Alzetani, Jennifer Richards, Lydia Scarlett, Papawadee Ingram, Serena Chee, Silvia Austin, Amrita Bajaj, Apostolos Nakas, Azmina Sodha-Ramdeen, Dean A. Fennell, Keng Ang, Mohamad Tufail, Mohammed Fiyaz Chowdhry, Molly Scotland, Rebecca Boyles, Sridhar Rathinam, Claire Wilson, Domenic Marrone, Sean Dulloo, Angeles Montero, Elaine Smith, Eustace Fontaine, Felice Granato, Helen Doran, Juliette Novasio, Kendadai Rammohan, Leena Joseph, Paul Bishop, Rajesh Shah, Stuart Moss, Vijay Joshi, Hugo J. W. L. Aerts, Tom L. Kaufmann, Roland F. Schwarz, Judit Kisistok, Mateo Sokac, Miklos Diossy, Zoltan Szallasi, Krijn Dijkstra, Yinyin Yuan, Crispin T. Hiley, Kevin Litchfield, Nicholas McGranahan, Mariam Jamal-Hanjani, Charles Swanton, George Kassiotis, CAPTURE Consortium, Annika Fendler, Lewis Au, Scott T. C. Shepherd, Nadia Yousaf, Fiona Byrne, Laura Amanda Boos, Benjamin Shum, Camille L. Gerard, Andreas M. Schmitt, Christina Messiou, David Cunningham, Ian Chau, Naureen Starling, Nicholas Turner, Liam Welsh, Robin L. Jones, Joanne Droney, Susana Banerjee, Kate C. Tatham, Shaman Jhanji, Kevin Harrington, Alicia Okines, Alison Reid, Kate Young, Andrew J. S. Furness, Lisa Pickering, Emma Nicholson, Sacheen Kumar, Katalin A. Wilkinson, Anthony Swerdlow, Robert J. Wilkinson, James Larkin, Samra Turajlic, George Kassiotis, Crispin T. Hiley, Kevin Litchfield, Nicholas McGranahan, Mariam Jamal-Hanjani, James Larkin, Se-Hoon Lee, Samra Turajlic, Charles Swanton, Julian Downward, George Kassiotis

**Affiliations:** 1 Retroviral Immunology Laboratory, The Francis Crick Institute, London, UK; 2 Oncogene Biology Laboratory, The Francis Crick Institute, London, UK; 3 Cancer Evolution and Genome Instability Laboratory, The Francis Crick Institute, London, UK; 4 Adult Stem Cell Laboratory, The Francis Crick Institute, London, UK; 5 Division of Hematology-Oncology, Department of Medicine, Samsung Medical Center, Sungkyunkwan University School of Medicine, Seoul, Republic of Korea; 6 Cancer Research UK Lung Cancer Centre of Excellence, University College London Cancer Institute, London, UK; 7 Cancer Metastasis Laboratory, University College London Cancer Institute, London, UK; 8 Department of Cellular Pathology, University College London Hospitals, London, UK; 9 Department of Pathology, ZAS Hospitals, Antwerp, Belgium; 10 Division of Research, Peter MacCallum Cancer Centre, Melbourne, Queensland Australia; 11 Bioinformatics and Biostatistics Facility, The Francis Crick Institute, London, UK; 12 Cancer Dynamics Laboratory, The Francis Crick Institute, London, UK; 13 Renal and Skin Units, The Royal Marsden Hospital, London, UK; 14 Cancer Genome Evolution Research Group, Cancer Research UK Lung Cancer Centre of Excellence, University College London Cancer Institute, London, UK; 15 Department of Health Sciences and Technology, Samsung Advanced Institute of Health Sciences and Technology, Sungkyunkwan University, Seoul, Republic of Korea; 16 National Heart and Lung Institute, Imperial College London, London, UK; 17 Advanced Sequencing Facility, The Francis Crick Institute, London, UK; 18 Lung Unit, The Royal Marsden Hospital, London, UK; 19 Division of Clinical Studies, The Institute of Cancer Research, London, UK; 20 Cancer Research UK and University College London Cancer Trials Centre, London, UK; 21 Tumour Immunogenomics and Immunosurveillance Laboratory, University College London Cancer Institute, London, UK; 22 Department of Oncology, University College London Hospitals, London, UK; 23 Melanoma and Kidney Cancer Team, The Institute of Cancer Research, London, UK; 24 Department of Infectious Disease, Faculty of Medicine, Imperial College London, London, UK; 25 Bill Lyons Informatics Centre, University College London Cancer Institute, London, UK; 26 University College London Cancer Institute, London, UK; 27 Computational Cancer Genomics Research Group, University College London Cancer Institute, London, UK; 28 Immune Regulation and Tumour Immunotherapy Group, Cancer Immunology Unit, Research Department of Haematology, University College London Cancer Institute, London, UK; 29 Department of Histopathology, Royal Brompton and Harefield Hospitals, Guy’s and St Thomas’ NHS Foundation Trust, London, UK; 30 Academic Division of Thoracic Surgery, Imperial College London, London, UK; 31 Royal Brompton and Harefield Hospitals, Guy’s and St Thomas’ NHS Foundation Trust, London, UK; 32 Cancer Genomics Laboratory, The Francis Crick Institute, London, UK; 33 Medical Genomics, University College London Cancer Institute, London, UK; 34 Integrative Cancer Genomics Laboratory, Department of Oncology, KU Leuven, Leuven, Belgium; 35 VIB–KU Leuven Center for Cancer Biology, Leuven, Belgium; 36 Department of Genetics, The University of Texas MD Anderson Cancer Center, Houston, TX USA; 37 Department of Genomic Medicine, The University of Texas MD Anderson Cancer Center, Houston, TX USA; 38 Cancer Immunology Unit, Research Department of Haematology, University College London Cancer Institute, London, UK; 39 Department of Haematology, University College London Hospitals, London, UK; 40 Centre for Medical Image Computing, Department of Medical Physics and Biomedical Engineering, University College London, London, UK; 41 Department of Medical Physics and Bioengineering, University College London Cancer Institute, London, UK; 42 Department of Medical Physics and Biomedical Engineering, University College London, London, UK; 43 Department of Radiology, University College London Hospitals, London, UK; 44 UCL Respiratory, Department of Medicine, University College London, London, UK; 45 Department of Thoracic Medicine, University College London Hospitals, London, UK; 46 Lungs for Living Research Centre, UCL Respiratory, University College London, London, UK; 47 Department of Thoracic Surgery, University College London Hospital NHS Trust, London, UK; 48 Experimental Histopathology, The Francis Crick Institute, London, UK; 49 Independent Cancer Patients’ Voice, London, UK; 50 Institute of Nuclear Medicine, Division of Medicine, University College London, London, UK; 51 Institute of Structural and Molecular Biology, University College London, London, UK; 52 Experimental Oncology, Institute for Oncology and Radiology of Serbia, Belgrade, Serbia; 53 Royal Free Hospital, Royal Free London NHS Foundation Trust, London, UK; 54 The Francis Crick Institute, London, UK; 55 The Institute of Cancer Research, London, UK; 56 The Whittington Hospital NHS Trust, London, UK; 57 University College London Hospitals, London, UK; 58 University College London, London, UK; 59 Aberdeen Royal Infirmary NHS Grampian, Aberdeen, UK; 60 Birmingham Acute Care Research Group, Institute of Inflammation and Ageing, University of Birmingham, Birmingham, UK; 61 Cancer Research Centre, University of Leicester, Leicester, UK; 62 Cancer Research UK Beatson Institute, Glasgow, UK; 63 Pathology Department, Queen Elizabeth University Hospital, NHS Greater Glasgow and Clyde, Glasgow, UK; 64 School of Cancer Sciences, University of Glasgow, Glasgow, UK; 65 Queen Elizabeth University Hospital, Glasgow, UK; 66 Cancer Research UK Lung Cancer Centre of Excellence, University of Manchester, Manchester, UK; 67 Cancer Research UK Manchester Institute Cancer Biomarker Centre, University of Manchester, Manchester, UK; 68 The Christie NHS Foundation Trust, Manchester, UK; 69 Division of Infection, Immunity and Respiratory Medicine, University of Manchester, Manchester, UK; 70 Wythenshawe Hospital, Manchester University NHS Foundation Trust, Wythenshawe, UK; 71 Department of Medical Oncology, Aberdeen Royal Infirmary NHS Grampian, Aberdeen, UK; 72 University of Aberdeen, Aberdeen, UK; 73 Department of Oncology, University Hospital Southampton NHS Foundation Trust, Southampton, UK; 74 Department of Pathology, Aberdeen Royal Infirmary NHS Grampian, Aberdeen, UK; 75 Division of Cancer Sciences, The University of Manchester and The Christie NHS Foundation Trust, Manchester, UK; 76 Golden Jubilee National Hospital, Clydebank, UK; 77 Institute of Immunology and Immunotherapy, University of Birmingham, Birmingham, UK; 78 University Hospital Birmingham NHS Foundation Trust, Birmingham, UK; 79 Liverpool Heart and Chest Hospital, Liverpool, UK; 80 Manchester Cancer Research Centre Biobank, Manchester, UK; 81 NHS Greater Glasgow and Clyde, Glasgow, UK; 82 Princess Alexandra Hospital, The Princess Alexandra Hospital NHS Trust, Harlow, UK; 83 Royal Surrey Hospital, Royal Surrey Hospitals NHS Foundation Trust, Guilford, UK; 84 University of Surrey, Guilford, UK; 85 Sheffield Teaching Hospitals NHS Foundation Trust, Sheffield, UK; 86 Singleton Hospital, Swansea Bay University Health Board, Swansea, UK; 87 University Hospital Southampton NHS Foundation Trust, Southampton, UK; 88 University Hospitals of Leicester NHS Trust, Leicester, UK; 89 University of Leicester, Leicester, UK; 90 Artificial Intelligence in Medicine (AIM) Program, Mass General Brigham, Harvard Medical School, Boston, MA USA; 91 Department of Radiation Oncology, Brigham and Women’s Hospital, Dana-Farber Cancer Institute, Harvard Medical School, Boston, MA USA; 92 Radiology and Nuclear Medicine, CARIM & GROW, Maastricht University, Maastricht, the Netherlands; 93 Berlin Institute for Medical Systems Biology, Max Delbrück Center for Molecular Medicine in the Helmholtz Association (MDC), Berlin, Germany; 94 Berlin Institute for the Foundations of Learning and Data (BIFOLD), Berlin, Germany; 95 Institute for Computational Cancer Biology, Center for Integrated Oncology (CIO), Cancer Research Center Cologne Essen (CCCE), Faculty of Medicine and University Hospital Cologne, University of Cologne, Cologne, Germany; 96 Bioinformatics Research Centre, Aarhus University, Aarhus, Denmark; 97 Department of Clinical Medicine, Aarhus University, Aarhus, Denmark; 98 Department of Molecular Medicine, Aarhus University Hospital, Aarhus, Denmark; 99 Computational Health Informatics Program, Boston Children’s Hospital, Boston, MA USA; 100 Danish Cancer Society Research Center, Copenhagen, Denmark; 101 Department of Physics of Complex Systems, ELTE Eötvös Loránd University, Budapest, Hungary; 102 Department of Bioinformatics, Semmelweis University, Budapest, Hungary; 103 Department of Molecular Oncology and Immunology, The Netherlands Cancer Institute, Amsterdam, the Netherlands; 104 Oncode Institute, Utrecht, the Netherlands; 105 The University of Texas MD Anderson Cancer Center, Houston, TX USA; 106 Department of Radiology, The Royal Marsden NHS Foundation Trust, London, UK; 107 Gastrointestinal Unit, The Royal Marsden NHS Foundation Trust, London, UK; 108 Breast Unit, The Royal Marsden NHS Foundation Trust, London, UK; 109 Breast Cancer Now Toby Robins Breast Cancer Research Centre, The Institute of Cancer Research, London, UK; 110 Neuro-oncology Unit, The Royal Marsden NHS Foundation Trust, London, UK; 111 Sarcoma Unit, The Royal Marsden NHS Foundation Trust, London, UK; 112 Palliative Medicine, The Royal Marsden NHS Foundation Trust, London, UK; 113 Gynaecology Unit, The Royal Marsden NHS Foundation Trust, London, UK; 114 Anaesthetics, Perioperative Medicine and Pain Department, The Royal Marsden NHS Foundation Trust, London, UK; 115 Department of Surgery and Cancer, Imperial College London, London, UK; 116 Head and Neck Unit, The Royal Marsden NHS Foundation Trust, London, UK; 117 Targeted Therapy Team, The Institute of Cancer Research, London, UK; 118 Acute Oncology Service, The Royal Marsden NHS Foundation Trust, London, UK; 119 Uro-oncology Unit, The Royal Marsden NHS Foundation Trust, London, UK; 120 Haemato-oncology Unit, The Royal Marsden NHS Foundation Trust, London, UK; 121 Tuberculosis Laboratory, The Francis Crick Institute, London, UK; 122 Division of Genetics and Epidemiology and Division of Breast Cancer Research, The Institute of Cancer Research, London, UK; 123 Wellcome Center for Infectious Disease Research in Africa, University of Cape Town, Cape Town, Republic of South Africa; 124 Present Address: Department of Molecular Medicine, Aarhus University Hospital, Aarhus, Denmark; 125 Present Address: Department of Clinical Medicine, Aarhus University, Aarhus, Denmark; 126 Present Address: Bioinformatics Research Centre, Aarhus University, Aarhus, Denmark; 127 Present Address: Cancer Stem Cell Laboratory, Institute of Cancer Research, London, UK; 128 Present Address: Division of Cancer, Department of Surgery and Cancer, Imperial College, London, UK; 129 Present Address: CRUK Convergence Science Centre, Imperial College, London, UK

**Keywords:** Non-small-cell lung cancer, Tumour immunology, Cancer genomics

Retraction to: *Nature* 10.1038/s41586-023-05771-9

The authors have retracted this article. An investigation by The Francis Crick institute found concerns regarding some of the data presented in the figures, specifically:

1. The A549 cell binding data in Fig. 5c are manipulated and could not be reproduced.

2. The antibody-dependent cellular cytotoxicity (ADCC) experiments shown in Fig. 5d and 5e may contain manipulated data.

3. The integrity of the source data for the B cell and antibody quantitation in Fig. 3c and Extended Data Fig. 5c could not be verified.

The Francis Crick Institute’s investigation found that co-first author Kevin Ng was responsible for these analyses.

The investigation recommended that the paper be retracted. A statement concerning the investigation is available on The Francis Crick Institute’s website (https://www.crick.ac.uk/about-us/our-approach-to-science/research-integrity/2025-research-integrity-news).

The authors apologize to the scientific community for any confusion and misunderstanding that may have arisen. They are committed to publishing a revised and properly validated research report in the near future.

There is no indication that the investigation’s findings impact the integrity of the TRACERx and CAPTURE clinical and genetic data associated with this work.

